# Up Close and Personal with an Internal-Membrane Virus

**DOI:** 10.1371/journal.pbio.1001668

**Published:** 2013-09-24

**Authors:** Caitlin Sedwick

**Affiliations:** Freelance Science Writer, San Diego, California, United States of America

Viruses have evolved a multitude of strategies for infiltrating their genome into host cells. Enveloped viruses such as herpesvirus and poxviruses wrap their genetic payload within a protein shell, or capsid, which itself is swathed in a lipid membrane that can fuse with a target cell's outer membrane. Other viruses, such as tailed bacteriophages, use a more complex mechanism: they inject their DNA into a host cell through a rigid tail that extends from their capsid. Yet another bacteriophage, an internal-membrane virus (and the best-studied example of this class of viruses) called PRD1, develops an infecting tail upon contact with a prospective host cell. Very little is known about this fascinating process—a deficit that Bibiana Peralta, Nicola G.A. Abrescia, and colleagues set out to address in their paper published in this month's *PLOS Biology*.

**Figure 1:**
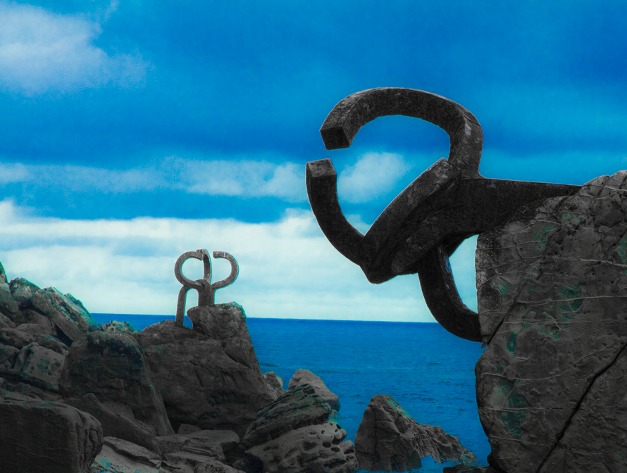
Bacteriophage PRD1 infects cells by shuttling its genome via a protruding tube that bears a striking resemblance to these extraordinary sculptures located not far from the Abrescia lab. Sculpture credit: *Wind Comb XV*, 1976. City of San Sebastian, Eduardo Chillida. Image credit: D. Gil-Carton.

The large class of internal-membrane viruses includes some members that attack eukaryotic cells and others that target archaea or bacteria. Like other internal-membrane viruses, PRD1's genome is contained within a lipid bilayer that sits beneath a proteinaceous capsid. PRD1's capsid is shaped like an icosahedron, with twenty triangular faces and twelve vertices; eleven of those vertices are structurally identical, while the twelfth is unique because it contains special proteins that help package the viral genome inside the capsid. Previous studies had shown that PRD1's tail forms from the internal lipid membrane and extends out through the capsid at one of its vertices. But until now no one had looked closely at how this takes place.

To get a good look at PRD1 tail formation, Peralta and colleagues used a variant of cryo-electron microscopy (cryo-EM) called cryo-electron tomography (cryo-ET). In cryo-EM, an electron beam is passed through a flash-frozen biological sample, and the scattering of this beam is used to generate a 2D image of sample contents. In cryo-ET, the sample is placed in a rotatable holder and is imaged from multiple angles, allowing for the reconstruction of a 3D image (tomogram). Because biological samples are very delicate, it's necessary to use a very low-power electron beam to avoid altering them. This inevitably results in a “noisy” 3D image that can be difficult to interpret. One way around this problem is to take several—often hundreds—of tomograms of different viruses in a sample, then average them together in a process called subtomogram averaging.

Using these approaches, Peralta et al. obtained 3D images of tail-forming PRD1 of unprecedented clarity. The images showed that the first step in tail formation is the removal of capsid proteins from some of the viral vertices, an uncapping that exposes the internal membrane to the exterior environment. The authors propose that the resulting change in osmolarity induces a redistribution of viral proteins present in the internal membrane, which then causes the tail to begin forming.

In agreement with earlier studies, Peralta and colleagues observed that tails extrude through one of the vertices in the viral capsid. In some images, immunolabeling showed components of the unique vertex marked in intact particles but not in particles with the extruding tail, as if the growing tail had displaced them. This led the authors to suggest that the tail may extrude through the unique vertex. But because it isn't yet known what the unique vertex looks like when it is intact, it wasn't possible to tell for sure.

To explore how the tail might be structured, Peralta et al. examined end-on cross-sections of viral tails. These analyses determined that the tails are hollow tubes of just the right diameter to allow passage of a single copy of the viral genome. From their thickness, it seems likely that the walls of these tubes consist of a lipid bilayer scaffolded by viral membrane–associated proteins. Just what that scaffold might look like is an open question, but the authors inferred that it might be either a helical or a linear structure because different cross-sections showed different shape profiles. In any case, once the tail has grown through the capsid vertex, it continues to extend. As more membrane is incorporated into the tail, the membrane-enclosed area remaining inside the capsid shell shrinks—a phenomenon that's frequently correlated with transition of the viral genome from the capsid space into the tail.

From there, the last step is inoculation of the target cell with viral DNA. Strikingly, Peralta et al. captured viruses in the act of penetrating bacterial membranes by perforating both the inner and outer membrane layers with their tails. Within 30 minutes of incubation with bacteria, the virus has achieved injection of its genomic payload into the host. But that isn't the end of this story; future studies will explore how these viruses activate and assemble their tails, which could help us understand analogous structures in other organisms.

**Peralta B, Gil-Carton D, Castaño-Díez D, Bertin A, Boulogne C, et al. (2013) Mechanism of Membranous Tunnelling Nanotube Formation in Viral Genome Delivery. doi:10.1371/journal.pbio.1001667**

